# Vitamin C Deficiency in Blood Samples of COVID-19 Patients

**DOI:** 10.3390/antiox11081580

**Published:** 2022-08-15

**Authors:** Tobias Sinnberg, Christa Lichtensteiger, Katharina Hill-Mündel, Christian Leischner, Heike Niessner, Christian Busch, Olga Renner, Nina Wyss, Lukas Flatz, Ulrich M. Lauer, Ludwig E. Hoelzle, Donatus Nohr, Markus Burkard, Luigi Marongiu, Sascha Venturelli

**Affiliations:** 1 Department of Dermatology, University Hospital Tuebingen, 72076 Tuebingen, Germany; 2 Department of Dermatology, Venereology and Allergology, Charité—Universitätsmedizin Berlin, 10117 Berlin, Germany; 3 Institute of Immunobiology, Kantonsspital St. Gallen, 9000 St. Gallen, Switzerland; 4 Institute of Nutritional Sciences, University of Hohenheim, 70599 Stuttgart, Germany; 5 Department of Nutritional Biochemistry, Institute of Nutritional Sciences, University of Hohenheim, 70599 Stuttgart, Germany; 6 Dermatologie zum Delfin, 8400 Winterthur, Switzerland; 7 Department of Internal Medicine VIII, University Hospital Tuebingen, 72076 Tuebingen, Germany; 8 German Cancer Consortium (DKTK), DKFZ Partner Site, 72076 Tuebingen, Germany; 9 Department of Livestock Infectiology and Environmental Hygiene, Institute of Animal Science, University of Hohenheim, 70599 Stuttgart, Germany; 10 Department of Vegetative and Clinical Physiology, Institute of Physiology, University of Tuebingen, 72074 Tuebingen, Germany

**Keywords:** COVID-19, vitamin plasma levels, ascorbate, calcidiol, retinol, α-tocopherol

## Abstract

Coronavirus disease 2019 (COVID-19) is the most notable pandemic of the modern era. A relationship between ascorbate (vitamin C) and COVID-19 severity is well known, whereas the role of other vitamins is less understood. The present study compared the blood levels of four vitamins in a cohort of COVID-19 patients with different severities and uninfected individuals. Serum concentrations of ascorbate, calcidiol, retinol, and α-tocopherol were measured in a cohort of 74 COVID-19 patients and 8 uninfected volunteers. The blood levels were statistically compared and additional co-morbidity factors were considered. COVID-19 patients had significantly lower plasma ascorbate levels than the controls (*p*-value < 0.001), and further stratification revealed that the controls had higher levels than fatal, critical, and severe COVID-19 cases (*p*-values < 0.001). However, no such trend was observed for calcidiol, retinol, or α-tocopherol (*p*-value ≥ 0.093). Survival analysis showed that plasma ascorbate below 11.4 µM was associated with a lengthy hospitalization and a high risk of death. The results indicated that COVID-19 cases had depleted blood ascorbate associated with poor medical conditions, confirming the role of this vitamin in the outcome of COVID-19 infection.

## 1. Introduction

In December 2019, an outbreak of atypical pneumonia was reported in the Wuhan region of China [1], and the World Health Organization declared it a pandemic within three months from the onset [2]. To date, the underlying disease, labeled as coronavirus disease 2019 (COVID-19), has infected over 440 million people and caused nearly 6 million deaths worldwide [3], with an estimated case-fatality rate ranging from 2.7 (95% confidence interval (CI): 2.3–3.1) to 10.0 (95% CI: 8.0–11.0) [4,5], corresponding to 68 deaths per 10,000 infections [6]. Several concomitant morbidities have been reported for COVID-19 such as smoking, age, and diabetes, although their role in the biology of the viral infection is still uncertain [7].

The COVID-19 etiological agent is the severe acute respiratory syndrome corona-virus 2 (SARS-CoV-2) (fam. *Coronaviridae*, subfam. *Orthocoronavirinae*, gen. Betacoronavirus, subgen. Sarbecovirus), an enveloped virus of about 100 nm in diameter with a capped and poly-adenylated positive-sense single-stranded RNA (ssRNA+) genome of 29 kb [8,9]. The envelope is decorated by spike (S) proteins involved in the viral attachment to the cellular surface receptors (carried out by the S1 subunit) and internalization (accomplished by the S2 subunit) [10]. The SARS-CoV-2 receptor is the angiotensin-converting enzyme 2 (ACE2), which is ubiquitously expressed in all human cells but particularly in the nasal and lung epithelium, explaining why the virus causes both upper and lower respiratory diseases together with the infection of several other organs [11]. The cellular response to viral infection might trigger an abnormal activation of T8 lymphocytes with consequent production of high levels of cytokines (hypercytokinemia or ‘cytokine storm’) that causes the acute respiratory distress syndrome (ARDS) responsible for the mortality in COVID-19 [12,13].

A well-balanced micronutrient intake is necessary for an effective immune response and reduction in adverse effects from infection, such as hospitalization in intensive care units [14,15]. Infection with SARS-CoV-2 is no different, and a healthy diet reduced the severity of ARDS [16]. Specifically, a balanced intake of both ascorbate (vitamin C) and calcidiol (vitamin D), among other micronutrients, is essential for the effectiveness of both innate and adaptive immunity [17,18].

Linus Pauling was the first to highlight the role of ascorbate in the prevention of the common cold, with etiological agents that include members of the *Coronaviridae* family [19,20]. Subsequent studies on rats proved that vitamin C reduced the deleterious effects of the cytokine storm by enhancing the expression of anti-oxidative enzymes (superoxide dismutase, catalase, and glutathione), while at the same time decreasing the expression of the pro-inflammatory cytokines TNF-α, interleukin (IL) 1β, and IL-23 and of anti-inflammatory IL-10 [20,21]. Ascorbate also reduced the proliferation of malignant cells through its anti-oxidant and anti-inflammatory activity [22,23]. Additionally, mouse studies showed that vitamin C improved the alveolar fluid clearance, reducing the attachment rate of the virus to its target cell [24]. Vitamin C supplementation could also be beneficial in attenuating the symptoms of post-viral fatigue associated with COVID-19 [25]. Other trials reported that taking 0.2 or 7 g of vitamin C daily reduced the duration of common cold symptoms by roughly 8% or 20%, respectively, although methodological problems were raised [26]. A thorough evaluation of the literature revealed that studies on the clinical effects of vitamin C were sparse [27]. Therefore, it is crucial to improve the understanding of vitamin C’s function in COVID-19.

Retinol (vitamin A) is central in the innate anti-viral response upon SARS-CoV-2 infection. The retinoic acid-inducible gene-I (RIG-I) proteins are vitamin A-dependent cytosolic receptors that recognize viral RNA and induce the expression of type I interferon (IFN-I) [28]. The massive amount of viral genomes produced by infected cells is thought to deplete the cellular retinol supply, shutting down IFN-I expression [29]. Consequently, there is a collapse of the innate immune response and hyper-activation of the pro-inflammatory T-helper 17 lymphocytes, triggering the cytokine storm [30]. Supporting this hypothesis, decreased plasma vitamin A was observed in hospitalized COVID-19 patients [31]. Calcidiol has a role similar to that of retinol in the innate immune response to SARS-CoV-2 [32,33,34]. It was suggested that COVID-19 associated mortality rate might be lower in countries where vitamin D supplementation is widespread, such as Norway or Finland [35]. In addition, vitamin D supplementation reduced the risk of infections with influenza virus and SARS-CoV-2 [36]. The association between α-tocopherol (vitamin E) and SARS-CoV-2 infection, instead, is less characterized.

In the present study, the plasma levels of four SARS-CoV-2 associated vitamins (ascorbate, retinol, calcidiol, and α-tocopherol) were measured in COVID-19 patients with varying disease severity and compared with the respective levels in healthy controls.

## 2. Materials and Methods

### 2.1. Patient Material and Clinical Data

Clinical data and blood samples of COVID-19 patients and healthy volunteers were collected from February to November 2020 at the Cantonal Hospital of St. Gallen, Switzerland. The study was approved by the local ethical committee (Swiss ethics protocol numbers 2020-01006, 2020-00566, and 2020-00646). Plasma samples were isolated from whole blood collected into heparin-containing tubes (BD Vacutainer CPT tubes, Becton Dickinson) following centrifugation at 1650× *g* for 20 min and cryo-stored at −80 °C. Sample aliquots of 65, 20, and 10 µL were prepared and used for the measurement of vitamins C and E, A, and D, respectively.

COVID-19 cases were classified as mild (no requirement for oxygen supplementation), severe (hospitalization due to SARS-CoV-2 infection together with non-invasive oxygen supplementation), critical (hospitalization due to SARS-CoV-2 infection with invasive oxygen supplementation), and fatal (hospitalization due to SARS-CoV-2 infection followed by demise), according to the Berlin definition [37].

### 2.2. Ascorbate Quantification

Aliquoted plasma samples were thawed at room temperature and subsequently centrifuged at 14,000× *g* for 5 min at 4 °C. The supernatant was transferred into new reaction tubes and either diluted 2:1 with deionized water for ascorbate analysis or 2:1 with aqueous 0.15 M tris(2-carboxyethyl)-phosphine hydrochloride solution to reduce dehydroascorbate for quantification of total ascorbate. Samples were centrifuged at 14,000× *g* for 5 min at 4 °C and instantly analyzed. As external standards aqueous ascorbate solutions were freshly prepared daily. For the stock solution, ascorbate was dissolved in cold 5% perchloric acid and diluted 1 + 1 with deionized water. The stock solution was further diluted with aqueous 0.15 M tris(2-carboxyethyl)-phosphine hydrochloride solution to reach final ascorbate concentrations of 2.0–75.0 µM. Standards were centrifuged at 14,000× *g* for 5 min at 4 °C before analysis. Plasma control (34.3 µM) was reconstituted with deionized water and stored at −80 °C. For analysis, plasma control was prepared in the same manner as the samples and measured daily before actual sample analysis for quality control. Sample, plasma control, and standard preparations were always performed on ice and protected from light, to avoid ascorbate degradation. Analysis was performed with HPLC, using a reversed-phase column (Reprosil-Pur 120 C18-AQ, 5 µm) with 25 mM aqueous sodium phosphate buffer (pH 3.0) as mobile phase. The flow rate was 1.0 mL/min and the injection volume was 20 µL. The HPLC system consisted of a DGU-20A3R degassing unit, two LC-20AT pumps, a SIL-20ACHT auto sampler (cooled at 4 °C), a CBM-20A communication module (Shimadzu GmbH, Duisburg, Germany). Detection was performed by using a Coulochem III electrochemical detector (ESA, Chelmsford, UK) and a high sensitivity analytical cell (Model 5011A, Thermo Scientific, Waltham, MA, USA) at −300 mV (E1, upstream) and +300 mV (E2, downstream).

### 2.3. Retinol and α-Tocopherol Quantification

A 20 µL aliquot of plasma was mixed with 100 µL ethanol and 100 µL of 2.35 µM retinyl acetate (dissolved in ethanol with butylated hydroxytoluene) as internal standard. The extraction of retinol and α-tocopherol was performed twice by adding respectively 1 mL of n-hexan and mixing for 10 s. The samples were subsequently centrifuged at 14,000× *g* for 5 min at room temperature. The hexane phase was transferred into a new reaction tube and rotary evaporated. The samples were redissolved in 100 µL buffer, composed of 80% acetonitrile and 20% tetrahydrofuran, before analysis. Standards were diluted with ethanol, measured photometrically, and again diluted with ethanol as indicated in the following: all-trans retinol (325 nm, ε = 52,770 L/mol × cm, 0.1–1.0 µM), all-trans retinol acetate (325 nm, ε = 51,180 L/mol × cm, 0.5–5.0 µM), and α-tocopherol (292 nm, ε = 3270 L/mol × cm, 0.1–15.0 µM). Standards were prepared once and stored at −80 °C until analysis. A vitamin A/E calibrator was measured within each HPLC run as a quality control. Sample, calibrator, and standard preparations were always performed light-protected. The column was a reversed-phase C18 column (ReproSil 80 ODS2, 250 mm × 4.6 mm, 3 µm). The mobile phase consisted of 5% deionized water and 95% of a mixture composed of 82% acetonitrile, 15% 1,4-dioxan, and 3% 30 mM ammonium acetate solution. Ammonium acetate solution was prepared with aqueous methanol (50% methanol/50% deionized water, v/v). The flow rate was 1.5 mL/min, and the analysis time was 25 min. The injection volume was 50 µL. For retinol and α-tocopherol analysis, the same HPLC system was used as for ascorbate analysis. Detection was performed by using a fluorescence detector RF-20A (Shimadzu GmbH, Duisburg, Germany). For simultaneous detection of retinol and α-tocopherol, the settings of the fluorescence detector were adjusted during the analysis as follows: retinol detection: 0.00–6.79 min, excitation 325 nm, emission 480 nm, sensitivity high, gain 4×; α-tocopherol detection: 6.80–25.00 min, excitation 298 nm, emission 328 nm, sensitivity medium, and gain 4×. The column was rinsed after 20 min for 2 min with 100% tetrahydrofuran. The autosampler was rinsed with 100% acetonitrile.

### 2.4. Calcidiol Quantification

Detection of calcidiol was obtained by competitive sandwich ELISA and conducted in accordance with the instructions of the manufacturer (PN 6411, EUROIMMUN, Lubeck, Germany). Briefly, 10 µL of plasma were diluted in 260 µL of biotin-labelled 25-OH vitamin D, and 100 µL of the mixture were added to microplate wells coated with monoclonal anti-25-OH vitamin D peroxidase-conjugated antibodies for 2 h. Unbound 25-OH vitamin D was removed by washing with washing buffer. Detection of bound biotin-labeled 25-OH vitamin D was obtained by incubation with tetramethylbenzidine (TMB) for 5 min. Optical density was measured using a microplate reader (TriStar3, Berthold, Germany) at the absorption wavelength 450 nm (with 620 nm as reference). Results were calculated using a standard curve prepared with the calibrators included in the kit. Each reaction was carried out in duplicates.

### 2.5. Data and Statistical Analysis

All chromatograms were recorded and analyzed using software LabSolutions ver. 5.71 (Shimadzu Deutschland GmbH, Duisburg, Germany). Statistical analysis was performed with R ver. 4.0. Pairs of groups were compared by either unpaired Student’s *t*-test or Mann–Whitney U test. Multiple groups were compared with either the Kruskal–Wallis H test or ANOVA, both with Bonferroni correction. Assessment of the normality of the sample distribution was performed with the Anderson–Darling test. Correlation between variables was obtained by Pearson’s product moment r. Receiver-operating characteristics (ROC) analysis was carried out with the R packages Epi and ROC [38]. Survival analysis, chi-squared (χ²) test, and Mantel–Haenszel hazard ratio (HR) were performed with GraphPad Prism ver. 9.3 (GraphPad Software, San Diego, CA, USA).

## 3. Results

The present investigation measured the blood concentration of four vitamins in a cohort of COVID-19 patients characterized by medical co-morbidities (*n* = 74) and uninfected volunteers (*n* = 8). The age of the whole cohort under evaluation is reported in Table 1. The clinical characteristics of cases are reported in Table 2. Measurements of the plasma vitamins and quantitative clinical characteristics are reported in Table 3.

**Figure 1:**
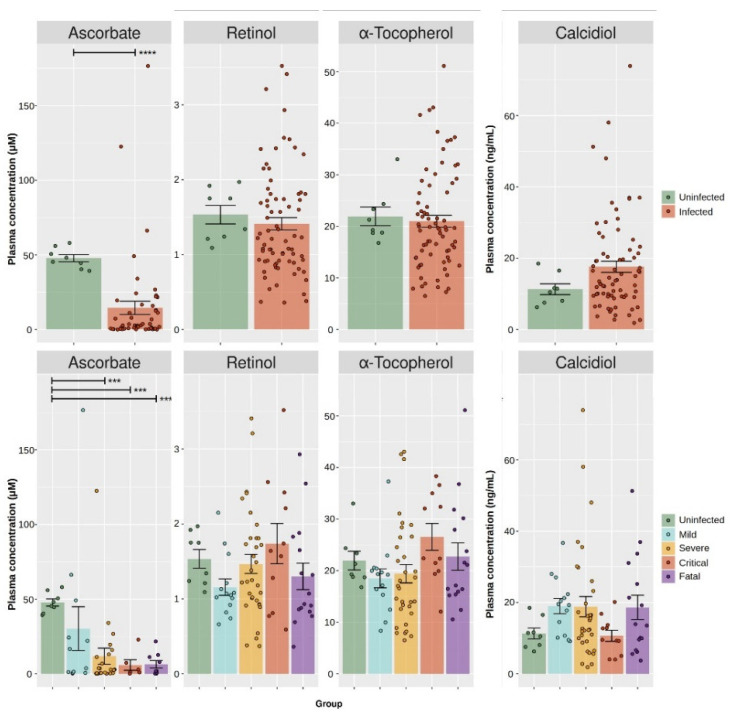
Measurement of plasma vitamins in COVID-19 patients and healthy controls. Plasma concentrations of selected vitamins. Take note of how two samples had ascorbate levels greater than 100 µM, setting them apart from the others; their removal did not change the reported statistical trends. Upper panel. Comparison of ascorbate, retinol, α-tocopherol, and calcidiol between healthy controls (uninfected) and COVID-19 cases (infected). Lower panel. Stratification of plasma ascorbate, retinol, α-tocopherol, and calcidiol by COVID-19 disease grade. Statistical significance: *p*-value < 0.001 (***), *p*-value < 0.0001 (****).

COVID-19 cases showed a significant decrease (Mann–Whitney U test *p*-value < 0.001) of ascorbate (median = 2.8 µM, interquartile range (IQR) = 0.5–15.2) in comparison to the controls (median = 46.7 µM, IQR = 43.5–51.9), but neither calcidiol, retinol, nor α-tocopherol showed significant differences (Mann–Whitney U test *p*-value ≥ 0.181; Figure 1, Table 3). Further stratification by disease severity confirmed a significant difference in total ascorbate levels between healthy controls and mild (median = 10.2 µM, IQR = 1.1–30.5), severe (median = 2.8 µM, IQR = 0.4–12.1), critical (median = 2.0 µM, IQR = 1.0–6.2), and fatal (median = 1.8 µM, IQR = 0.6–11.2) COVID-19 cases (Kruskal–Wallis H test *p*-value = 0.002; significant cut-off for pair testing: 0.001). Pair-wise analysis confirmed differences in plasma ascorbate between the controls and severe, critical, and fatal (*p*-value < 0.001 in all instances) COVID-19 cases, but not against mild cases (*p*-value = 0.039). Such a trend was, instead, not observed for calcidiol, retinol, or α-tocopherol (Kruskal–Wallis H test *p*-value ≥ 0.093). Two COVID-19 samples had plasma ascorbate above 100 µM and were flagged as outliers. Even after the removal of these samples, the group differences reported above were still significant (*p*-values < 0.001). To reduce the impact of these two outliers, further analysis was performed on the logarithm-transformed data.

**Figure 2:**
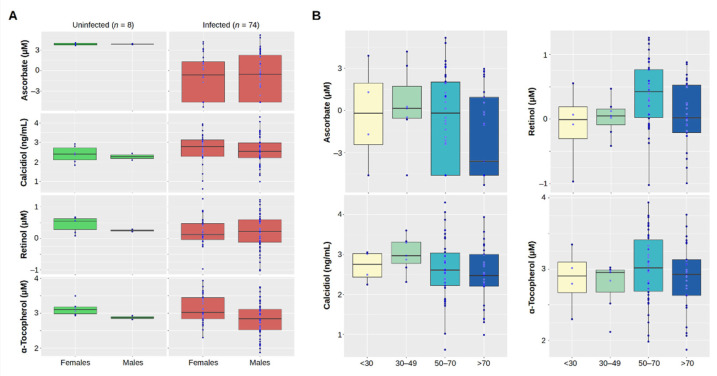
Stratification of plasma vitamins by gender and age. (**A**) Comparison of the natural logarithm of plasma ascorbate, calcidiol, retinol, and α-tocopherol between healthy controls (uninfected) and COVID-19 cases (infected) stratified by gender. (**B**) Stratification of plasma ascorbate, calcidiol, retinol, and α-tocopherol in the COVID-19 patients by age group. To reduce the impact of two samples with plasma ascorbate levels above 100 µM, the measured values are expressed as the natural logarithm (*ln*). This type of transformation reduces the spread of the data, while also assisting in meeting the assumptions of a statistical inference and improving their interpretation.

Further stratification by gender (Figure 2A) did not show significant differences between males (*n* = 49) and females (*n* = 33), neither for COVID-19 cases nor the controls (*t*-test *p*-value ≥ 0.108), with the exception of α-tocopherol: this vitamin was significantly lower in both male controls (2.9 ± 0.1 *ln*(µM)) than in female controls (3.1 ± 0.2 *ln*(µM), *p*-value = 0.047) and male cases (2.8 ± 0.5 *ln*(µM)) than in female cases (3.1 ± 0.4 *ln*(µM), *p*-value = 0.006). Vitamin blood levels were stratified by age group (Figure 2B). Although blood levels of ascorbate were lower in COVID-19 cases older than 70 years, there were no statistical differences between the age groups (ANOVA *p*-value = 0.290). Similarly, there were no differences in the age groups for calciferol and α-tocopherol (ANOVA *p*-value ≥ 0.355). Group differences hinted to a higher blood retinol in the age-bin 50–70 years (ANOVA *p*-value = 0.036). Although there was a significant difference between the age-bin 30–49 against the both the bins 50–70 (t-test *p*-value = 0.021) and over 70 (t-test *p*-value = 0.022), it was not above the Bonferroni-corrected level of 0.008. There was no particular correlation between ascorbate and the other vitamins (*p*-value ≥ 0.104), although α-tocopherol and retinol did show a significant positive correlation: *r* = 0.482 (95% CI: 0.285–0.640), *p*-value < 0.001 (Supplementary Figure S1).

**Figure 3:**
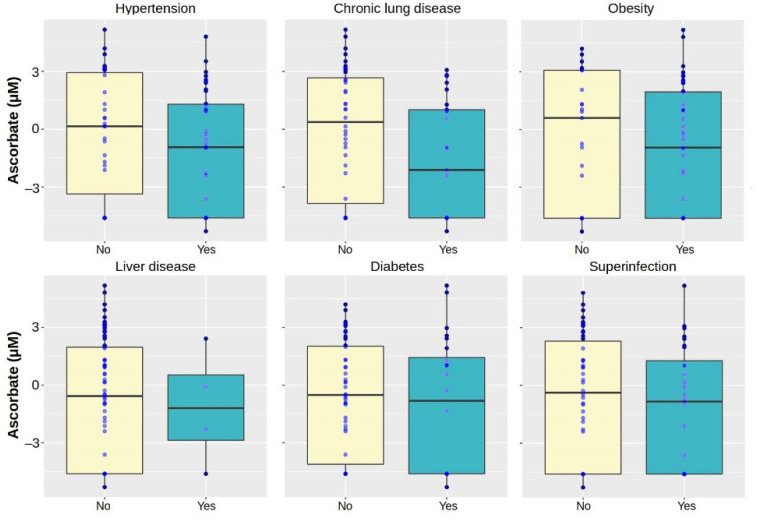
Stratification of plasma ascorbate in the COVID-19 patients by selected clinical features. To reduce the impact of two samples with plasma ascorbate levels above 100 µM, the measured values are expressed as the natural logarithm (*ln*). This type of transformation reduces the spread of the data, while also assisting in meeting the assumptions of a statistical inference and improving their interpretation.

Plasma ascorbate was stratified by selected clinical features (Figure 3). There were no significant differences in ascorbate between presence (−1.3 ± 3.1 *ln*(µM)) or absence (−0.2 ± 3.2 *ln*(µM)) of hypertension (*p*-value = 0.146), chronic lung disease (*p*-value = 0.066), liver failure (*p*-value = 0.862), diabetes (*p*-value = 0.535), obesity (*p*-value = 0.502), or superinfection (*p*-value = 0.398). Furthermore, the relationship between vitamin C and immune response was investigated by determining the Pearson’s product–moment correlation *r* between C-reactive protein (CRP) and the logarithm of the total blood ascorbate. High levels of CRP corresponded to low levels of ascorbate, albeit this correlation was non-significant (*r* = −0.141, *p*-value = 0.379).

**Figure 4:**
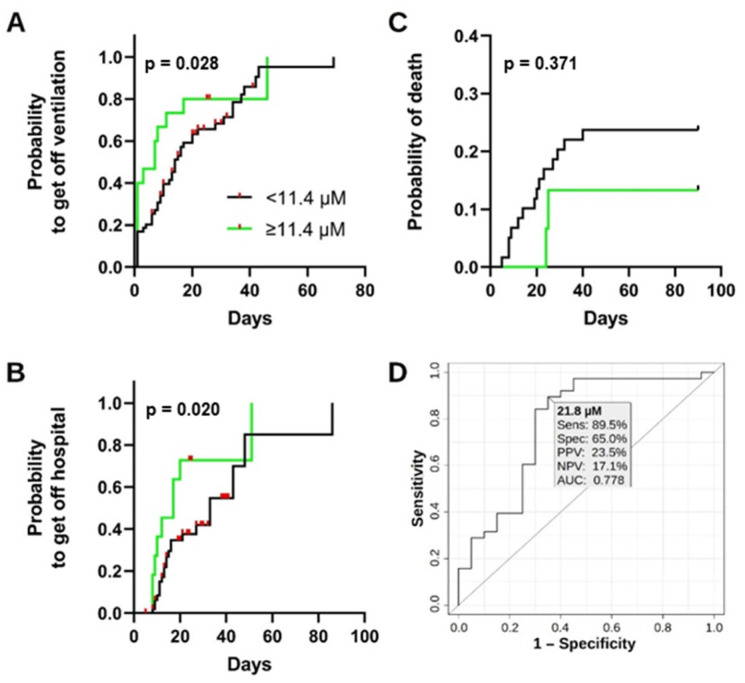
Survival analysis based on vitamin C levels in COVID-19 patients. (**A**) Time until the end of either ventilation time, (**B**) hospitalization, or (**C**) a COVID-19 related death event based on the widely accepted cut-off for vitamin C deficiency (plasma ascorbate of 11.4 µM) for the 74 COVID-19 cases. *p*-values are calculated by the Gehan-Breslow-Wilcoxon test. (**D**) ROC analysis for the differentiation of healthy controls and mild COVID-19 cases based on plasma ascorbate. The ROC curve is constructed by plotting the sensitivity against the false positive rate (1—specificity) at various threshold of plasma ascorbate. ROC analysis helps selecting cut-off points to separate two populations, in this case uninfected people and mild COVID-19 cases. A cut-off of 21.8 µM achieved a sensitivity (Sens.) of 89.5%, a specificity (Spec.) of 65.0%, a PPV of 23.5%, a NPV of 17.1%, and an AUC of 0.778. AUC, area under the curve; NPV, negative predictive value; PPV, positive predictive value; ROC, receiver-operating characteristics.

Survival analysis based on a plasma ascorbate (Figure 4A–C) showed that COVID-19 patients with plasma ascorbate below the widely accepted threshold for vitamin C deficiency of 11.4 µM [39,40,41,42] had a median period of ventilation of 14 days, compared to 7 days for patients with concentrations above this cut-off, resulting in an HR of 0.591 (95% CI: 0.281–1.246). Using the same cut-off, patients with low ascorbate had a median period of hospitalization of 33 days compared to 17 days for those with high ascorbate, corresponding to an HR of 0.393 (95% CI: 0.148–0.960). Cases with low total ascorbate displayed more death events within the first 21 days after presentation in the hospital due to COVID-19 (9/59 or 15%) than patients with normal plasma levels (0/15 or 0%). The HR for mortality was 1.722 (95% CI: 0.524–5.658) when comparing patients with low and high plasma ascorbate. However, in none of the cases was the plasma ascorbate sufficient to significantly differentiate these classes: the *χ*² test *p*-value was ≥0.060 in all instances. ROC analysis identified the plasma ascorbate cut-off of 36.7 µM to differentiate between healthy controls and COVID-19 patients, with a specificity of 100% and a sensitivity of 92.0% (data not shown). Healthy controls and mild COVID-19 cases could be separated from more severe COVID-19 outcomes by a plasma ascorbate cut-off of 21.8 µM, with a specificity of 65.0% and a sensitivity of 89.5% (Figure 4D).

## 4. Discussion

In this study, we demonstrated that plasma ascorbate is lower in patients with COVID-19 than in uninfected SARS-CoV-2 negative individuals. The vitamins quantified are all considered essential for the immune response to viral infections [43], thus the study aimed at assessing whether they could play a role in SARS-CoV-2 infection. The data gathered herein did not show a COVID-19 specific decrease in blood calciferol or retinol as previously reported for COVID-19 [31,44], whereas the data confirmed previous investigations reporting decreased plasma ascorbate in COVID-19 cases [45,46,47] and the absence of relation between calcidiol and COVID-19 severity [48].

So far, two other studies reported low ascorbate levels in COVID-19 cases. Indeed, according to a recent study, vitamin C levels were undetectable in more than 90% of COVID-19 patients with ARDS [43]. Moreover, an epidemiological study reported that up to 82% of critically ill adult COVID-19 patients with ARDS had low vitamin C levels [45,49]. Supporting the depletion of ascorbate in COVID-19 reported herein, and, in particular, the inverse relation between plasma concentration of this vitamin and COVID-19 severity, cases with clinical ascorbate deficiency (scurvy), defined as plasma levels below 11.4 µM, showed a higher death rate, ventilation time, and hospitalization than those with higher ascorbate levels.

It is estimated that up to 45% of the population in the United States is vitamin C deficient [50], and it has been reported that about one-tenth of Europeans suffer from deficiency of at least one vitamin [43]. Such a widespread vitamin deficiency overlaps with the SARS-CoV-2 pandemic and could help to explain the disease’s severity. Therefore, supplementation of vitamin C at high dosages (high-dose intravenous, HDIV) may be beneficial to maintain cellular homeostasis and an immune response. Several studies have reported how administration of vitamin C reduced the ARDS’ severity and fatality rate in COVID-19 [14,26,51]. For instance, COVID-19 patients receiving 11,000 mg per day demonstrated an increased lymphocyte count [52], 7000 mg per day reduced the incidence of systemic inflammatory syndrome [53], and 6000 mg per day increased the peripheral capillary oxygen saturation, thus reducing the need for forced ventilation [54]. Even just an intake of 500 mg per day significantly increased the production of anti-spike and neutralizing antibodies against SARS-CoV-2 [55].

The reasons for ascorbate’s beneficial effects during viral infection are multifaceted. The primary protective role of this vitamin is probably linked to its anti-inflammatory properties [56,57]. For instance, in severe COVID-19 patients, high-dose administration of ascorbate resulted in lower IL-6 levels compared to a placebo group [58]. In addition, the anti-oxidative properties of vitamin C decrease the impact of oxidative stress on the cellular homeostasis as well as the activation of the cytokine storm [59,60]. So ascorbate, among other reducing agents, can reduce the tocopheroxyl radical back to tocopherol and, thus, contribute to a kind of vitamin E recycling in vivo followed by a reinforcement of the cellular antioxidant capabilities, especially in lipophilic environments [61]. Likely, combinational supplementation of vitamin C and E could be beneficial in future preventive interventions. Ascorbate might even directly affect the viral life cycle, since it has been reported that this vitamin prevents the oxidation of thiol groups within ACE2, triggering a conformational change that reduces the efficiency of fusion step of SARS-CoV-2 infection [62,63,64].

Our study includes some limitations. Firstly, there was a high proportion of patients (79.7%) with undetectable plasma ascorbate, which might suggest an artifact due to degradation of this vitamin. However, samples were always processed quickly before freezing, and samples that could not be processed on the same day of collection were not included in the present analysis. Secondly, pre-infection vitamin blood levels were not available for the present study, so it could not be determined whether ascorbate deficiency was a cause or consequence of SARS-CoV-2 infection in the patients studied here. However, infections in general are known to cause a rapid decrease in plasma ascorbate levels [65], resulting in a lack of attenuation of the pro-inflammatory cell signaling pathway NF-κB as well as a decrease in the expression of molecules that counteract the harmful effects of reactive oxidative species (ROS), such as superoxide dismutase, catalase, and glutathione. It can be hypothesized that most of the cases included in the present study had normal ascorbate levels at baseline but lost them upon the progression of COVID-19, although prospective studies are needed to confirm this point. This study demonstrates the important role of vitamin C in the context of SARS-CoV-2 infection. Since three fat-soluble vitamins (vitamins A, D, and E) and only one water-soluble vitamin (vitamin C) were analyzed in the current work, it would be of great interest to determine values of other water-soluble representatives from the vitamin B complex as a next step and to evaluate possible correlations with SARS-CoV-2 infection. Besides the technical–analytical aspects of this work, it is further important to consider that initial vitamin blood levels as well as the patient’s lifestyle (e.g., exposure to sunshine, in the particular case of vitamin D), dietary habits, and nutritional status could have an impact on the onset of SARS-CoV-2 infection and its progress. In future prospective studies, collection of these information should be included into the study protocol to have a baseline for plasma vitamin levels. However, in clinical practice, this is difficult to perform in a non-interventional, observational-only study, as patients are usually treated in the clinic when they already have symptoms of disease.

The present study’s findings are valuable for medical interventions to reduce the COVID-19 burden of infection and the results corroborated the context of the literature regarding the association between vitamin C and SARS-CoV-2 infection. The role of vitamin measurement could be either preventive for or supportive to treat COVID-19 infections. In the former case, identifying healthy people with plasma ascorbate below specific levels could pinpoint who might develop high-grade COVID-19. Such levels could be based, for instance, on the 21 µM indicated by the ROC analysis provided herein (which is remarkably close to the level of 23 µM reported for hypovitaminosis [66]) or the accepted threshold of 11.4 µM for deficiency [39]. COVID-19 cases with low plasma ascorbate might likely progress to high-grade symptoms. In both cases, quantification of plasma ascorbate might prove an important implement in the management of COVID-19 cases. Therefore, regular supplementation of vitamin C might be beneficial for both preventing and curing the disease.

Only a few randomized clinical trials assessed the effectiveness of ascorbate in reducing adverse symptoms in COVID-19 patients. Some studies did not find any added value in supplementing ascorbate [54,58], whereas others had reported a quicker recovery when anti-inflammatory drugs, prophylactic antibiotics, or even traditional remedies were supplemented with ascorbate [67,68,69]. These conflicting results call for more research, which is also desirable given the possibility of new pandemics in the future, increasing the level of evidence for the employment of ascorbate in the treatment of COVID-19 and other respiratory diseases.

## 5. Conclusions

Vitamin C plays an important role in suppressing the pathological processes triggered by SARS-CoV-2, due to its pleiotropic physiological effects discussed above. Among others, it has immune-supportive, antiviral, anti-inflammatory, antioxidant, and antithrombotic properties [70,71]. Therefore, together with the here-described scurvy-like plasma levels of ascorbate, vitamin C, especially high-dose intravenous vitamin C, can be considered an important supportive component in the treatment of SARS-CoV-2 infections. Since pharmacokinetic mechanisms set natural limits to oral supplementation, intravenous administration should be considered for acute deficiency. However, it remains to be emphasized that the available study results show beneficial effects especially when administered early (before the onset of severe or even critical symptoms) and at doses of at least 100 mg/kg daily.

## Figures and Tables

**Table 1:** Stratification of patients and controls according to age and gender.

Parameter	Total (*n* = 82)	Males (*n* = 49)	Females (*n* = 33)
Age			
Less than 30 years	8 (9.76%)	0	8 (24.24%)
30–49 years	11 (13.41%)	4 (8.16%)	7 (21.21%)
50–70 years	38 (46.34%)	27 (55.10%)	11 (33.33%)
More than 70 years	25 (30.49%)	18 (36.73%)	7 (21.21%)
Controls	8 (9.76%)	2 (4.08%)	6 (18.18%)

**Table 2:** Stratification of patients according to clinical characteristics of COVID-19 symptoms.

Parameter	Total (*n* = 74)	Males (*n* = 47)	Females (*n* = 27)
SARS severity			
——Mild	14 (18.92%)	4 (8.51%)	10 (37.04%)
——Severe	33 (44.59%)	24 (51.06%)	9 (33.33%)
——Critical	11 (14.86%)	7 (14.89%)	4 (14.81%)
——Fatal	16 (21.62%)	12 (25.53%)	4 (14.81%)
Ventilation			
——None	14 (18.92%)	4 (8.51%)	10 (37.04%)
——Nasal	18 (24.32%)	11 (23.40%)	7 (25.93%)
	11 (14.86%)	8 (17.02%)	3 (11.11%)
——Noninvasive vent	11 (14.86%)	9 (19.15%)	2 (7.41%)
	15 (20.27%)	11 (23.40%)	4 (14.81%)
	5 (6.76%)	4 (8.51%)	1 (3.70%)
Use of corticosteroids	62 (83.78%)	45 (95.74%)	17 (62.96%)
Concomitant bacterial infections	28 (37.84%)	20 (42.55%)	8 (29.63%)
Kidney failure	14 (18.92%)	11 (23.40%)	3 (11.11%)
Sepsis	5 (6.76%)	5 (10.64%)	0
Pancreatitis	1 (1.35%)	1 (2.13%)	0
Coagulation failure	6 (8.11%)	4 (8.51%)	2 (7.41%)
Cardiac failure	6 (8.11%)	4 (8.51%)	2 (7.41%)
Liver failure	4 (5.41%)	4 (8.51%)	0
Other general symptoms	9 (12.16%)	7 (14.89%)	2 (7.41%)
Hematological disorders	16 (21.62%)	9 (19.15%)	7 (25.93%)
Diabetes	27 (36.49%)	18 (38.30%)	9 (33.33%)
Cancer	13 (17.57%)	7 (14.89%)	6 (22.22%)
Hypertension	43 (58.11%)	32 (68.09%)	11 (40.74%)
Obesity	49 (66.22%)	33 (70.21%)	16 (59.26%)
Chronic lung disease	34 (45.95%)	18 (38.3%)	16 (59.26%)

**Table 3:** Exploratory statistics of selected parameters for COVID-19 cases.

Parameter	Total	Males	Females
Age (years) *	65 (57–73)	66 (60–73)	65 (33–69)
Hospitalization (days) *	14 (8–24)	17 (11–27)	11 (0–17)
Ventilation (days) *	10 (4–21)	14 (7–26)	6 (0–13)
Retinol (µM) †	1.412 ± 0.714	1.431 ± 0.746	1.380 ± 0.668
α-tocopherol (µM) †	21.007 ± 9.771	18.850 ± 8.839	24.762 ± 10.335
Ascorbate, reduced (µM) *	2.777 (0.548–15.172)	2.777 (0.570–13.725)	2.703 (0.687–14.180)
Ascorbate, total (µM) *	5.686 (1.547–18.328)	7.882 (1.788–16.490)	2.600 (0.948–18.427)
Ascorbate, dehydro (µM) *	1.787 (0.394–3.984)	1.868 (0.435–4.059)	1.558 (0.428–3.121)
Calcidiol (ng/mL) †	17.589 ± 13.353	17.110 ± 13.994	18.424 ± 12.371
C-reactive protein (mg/L) †	175.047 ± 101.988	193.978 ± 103.064	130.211 ± 86.152

*: median (interquartile range). †: mean ± standard deviation.

## Data Availability

Not applicable.

## Supplementary Materials

The following supporting information can be downloaded at: https://www.mdpi.com/article/10.3390/antiox11081580/s1. Figure S1: Correlation of vitamins in COVID-19 patients.
