# Supplementary material for: Vitamin C Deficiency in Blood Samples of COVID-19 Patients
Source: Antioxidants (Basel). 2022 Aug 15;11(8):1580. doi: 10.3390/antiox11081580 (PMC9405075; doi:10.3390/antiox11081580)
Supplement: Supplementary file 1 [file antioxidants-11-01580-s001.zip › antioxidants-1846843-supplementary.pdf]

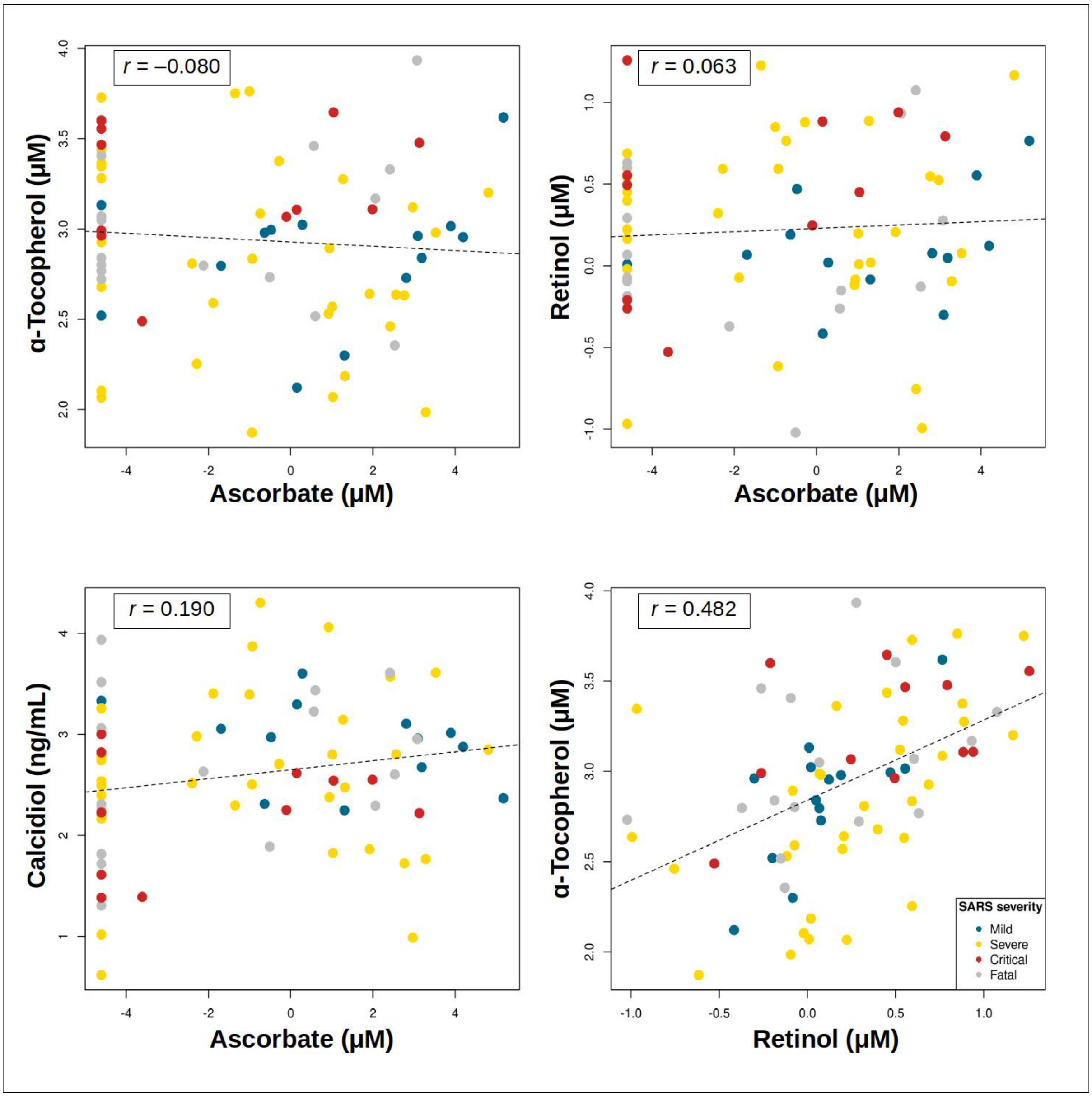

Figure S1. Correlation between plasma ascorbate, α-tocopherol, retinol, and calcidiol in the COVID-19 patients. The data are stratified by SARS severity. The Pearson's product moment coefficient r for each pair is depicted. This coefficient measures the magnitude of linear correlation between two sets of data: the more r is closed to unity, the more the two variables are linearly associated. The regression slope determines the sign of the correlation. SARS, severe acute respiratory syndrome.
